# Dipeptide repeat protein inclusions are rare in the spinal cord and almost absent from motor neurons in C9ORF72 mutant amyotrophic lateral sclerosis and are unlikely to cause their degeneration

**DOI:** 10.1186/s40478-015-0218-y

**Published:** 2015-06-25

**Authors:** Jorge Gomez-Deza, Youn-bok Lee, Claire Troakes, Matthew Nolan, Safa Al-Sarraj, Jean-Marc Gallo, Christopher E. Shaw

**Affiliations:** Department of Basic and Clinical Neurosciences, Maurice Wohl Clinical Neurosciences Institute, Institute of Psychiatry, Psychology and Neuroscience, Kings College London, London, SE5 8AF UK; London Neurodegenerative Disease Brain Bank, Institute of Psychiatry, Psychology and Neuroscience, King’s College London, London, SE5 8AF UK

**Keywords:** C9ORF72, Dipeptide repeat, TDP-43, Motor neuron, Amyotrophic lateral sclerosis

## Abstract

**Introduction:**

Cytoplasmic TDP-43 inclusions are the pathological hallmark of amyotrophic lateral sclerosis (ALS) and tau-negative frontotemporal lobar dementia (FTLD). The G_4_C_2_ repeat mutation in *C9ORF72* is the most common cause of ALS and FTLD in which, in addition to TDP-43 inclusions, five different di-peptide repeat (DPR) proteins have been identified. Di-peptide repeat proteins are translated in a non-canonical fashion from sense and antisense transcripts of the G_4_C_2_ repeat (GP, GA, GR, PA, PR). DPR inclusions are abundant in the cerebellum, as well as in the frontal and temporal lobes of ALS and FTLD patients and some are neurotoxic in a range of cellular and animal models, implying that DPR aggregation directly contributes to disease pathogenesis. Here we sought to quantify inclusions for each DPR and TDP-43 in ALS cases with and without the C9ORF72 mutation. We characterised the abundance of DPRs and their cellular location and compared this to cytoplasmic TDP-43 inclusions in order to explore the role of each inclusion in lower motor neuron degeneration.

**Results:**

Spinal cord sections from ten cases positive for the *C9ORF72* repeat expansion (ALS-C9+ve) and five cases that were not were probed by double immunofluorescence staining for individual DPRs and TDP-43. Inclusions immunoreactive for each of the DPRs were present in the spinal cord but they were rare or very rare in abundance (in descending order of frequency: GA, GP, GR, PA and PR). TDP-43 cytoplasmic inclusions were 45- to 750-fold more frequent than any DPR, and fewer than 4 % of DPR inclusions colocalized with TDP-43 inclusions. In motor neurons, a single cytoplasmic DPR inclusion was detected (0.1 %) in contrast to the 34 % of motor neurons that contained cytoplasmic TDP-43 inclusions. Furthermore, the number of TDP-43 inclusions in ALS cases with and without the *C9ORF72* mutation was nearly identical.

**Conclusions:**

For all other neurodegenerative diseases, the neurotoxic protein aggregates are detected in the affected population of neurons. TDP-43 cytoplasmic aggregation is the dominant feature of ALS spinal cord pathology irrespective of *C9ORF72* mutation status. The near absence of DPR inclusions in spinal cord motor neurons challenges their contribution to lower motor neuron degeneration in ALS-C9+ve cases.

**Electronic supplementary material:**

The online version of this article (doi:10.1186/s40478-015-0218-y) contains supplementary material, which is available to authorized users.

## Introduction

Amyotrophic lateral sclerosis (ALS) is a progressive neurodegenerative disease characterised by the loss of motor neurons in the brain and spinal cord causing progressive paralysis of limb and bulbar function and death due to respiratory failure within an average of 3 years from symptom onset. Cytoplasmic inclusions containing the transactive response DNA-binding protein (TDP-43) are the neuropathological hallmark of ~95 % of ALS cases and ~60 % of frontotemporal dementia (FTD) cases [1]. Expansion of an intronic hexanucleotide repeat (G_4_C_2_) in chromosome 9 open reading frame 72 (*C9ORF72*) is the most common genetic cause of ALS in people of European ancestry [2–4]. The number of pathological repeats varies from 300–3000 in ALS cases and contrasts with up to 30 repeats found in healthy controls.

The neurotoxic mechanisms of the *C9ORF72* expansion are unknown but three mechanisms have been proposed: loss of C9ORF72 protein function; toxicity due to RNA aggregation and toxicity due to repeat associated non-ATG (RAN) translation of dipeptide repeat (DPR) proteins. Although the G_4_C_2_ expansion does cause a decrease in *C9ORF72* mRNA, it is not clear that this leads to loss of the C9ORF72 protein and no null alleles or missense mutations in *C9ORF72* have been identified in ALS or FTD cases to support the loss of C9ORF72 protein function hypothesis. We, and others have shown that G_4_C_2_ repeats generate length-dependant RNase-resistant intranuclear RNA foci, which can sequester RNA binding proteins potentially leading to defective RNA processing [5]. Finally, the G_4_C_2_ repeat RNA form stable G-quadruplex secondary structures that can recruit the translation machinery [6] and generate five different DPRs; poly-GP, poly-GA and poly-GR from the sense G_4_C_2_ repeat strand and poly-PR, poly-PA and poly-GP from the antisense G_2_C_4_ repeat strand [7,8]. This last hypothesis has recently garnered much attention and is the one we wish to test in the context of human ALS tissues.

Antibodies to specific DPR proteins detect cytoplasmic aggregates of poly-GP, poly-GA and poly-PA and intranuclear aggregates of poly-PR and poly-GR in the cerebellum and frontal cortex of ALS and FTD patients [9,10]. DPR protein aggregates colocalize with p62 but not TDP-43 [9,10] and the connection between the *C9ORF72* mutation, DPR protein deposition and TDP-43 mislocalization is unknown. In the present study, we conducted a detailed comparison of the abundance and cellular location of the five DPR proteins and TDP-43 in the spinal cord of ten ALS-C9+ve cases. Using double immunofluorescence staining, we show that DPR protein aggregates are uncommon in the spinal cord of ALS-*C9+ve* cases, rarely co-localise with TDP-43, and are almost absent from motor neurons. The abundance of TDP-43 inclusions in ALS cases with or without a *C9ORF72* repeat expansion was broadly similar. Given that TDP-43 mislocalization is a recognised cause of lower motor neuron neurodegeneration, and given the lack of DPRs in motor neurons, it is difficult to implicate DPRs in this process.

## Materials and methods

### Cases

A total of 30 spinal cord sections were analysed for the presence of TDP-43 and all DPR inclusions by double immunofluorescence from ten ALS cases positive for *C9ORF72* intronic expansion (ALS-*C9+ve*) and 13 sections from five ALS cases negative for *C9ORF72*, *FUS* and *SOD1* mutations (365 sections in total). Sections of frontal lobe and cerebellar cortex from a case of C9FTD with abundant p62 pathology were chosen as a positive control for DPR staining. All cases were provided by the Medical Research Council London Neurodegenerative Diseases Brain Bank (Institute of Psychiatry, Psychology and Neuroscience, King’s College London). Samples were collected and distributed in accordance with local and national research ethics committee approvals. Expansion carriers were identified using repeat primed PCR [4] and all of the cases had previously been reported to show characteristic cerebellar p62 and TDP-43 pathology [11,12]. Details of the age, sex and post-mortem delay are recorded in Table 1 and show little difference between cases with and without *C9ORF72* mutations.

**Table 1:** Clinical Data of cases studied

Case	Age	Sex	PMD	Diagnosis
1	73	M	42	ALS-TDP
2	70	F	27	ALS-TDP
3	60	M	70	ALS-TDP
4	44	F	24	ALS-TDP
5	68	M	5	ALS-TDP
mean ± SEM	63 ± 5		33 ± 11	ALS-TDP
6	59	F	35	ALS-C9 + ve
7	70	M	38	ALS-C9 + ve
8	59	M	46	ALS-C9 + ve
9	43	F	69	ALS-C9 + ve
10	53	M	82	ALS-C9 + ve
11	70	M	60	ALS-C9 + ve
12	55	M	76	ALS-C9 + ve
13	58	M	11	ALS-C9 + ve
14	64	M	68	ALS-C9 + ve
15	51	M	64	ALS-C9 + ve
mean ± SEM	58 ± 2		55 ± 5	

*PMD* post mortem delay (hours)

### Motor neuron counting and double label immunofluorescence

Histological examination was performed on 7 μm sections prepared from formalin-fixed, paraffin –embedded tissue from spinal cords of all ALS cases. Sections were stained with hematoxylin and eosin to perform motor neuron counts, and sequential sections were processed for double label immunofluorescence for the DPRs and TDP-43. Prior to double immunofluorescence staining, paraffin was removed with xylene and all sections were rehydrated in an ethanol series (100, 95, and 70 %) for 3 min per step. Slides were incubated in 0.3 % Sudan black for 5 min to quench autofluorescence and washed with water. Antigen retrieval was carried out by microwaving for 6 min at maximum power and 12 min at medium power in 100 mM sodium citrate buffer (pH 6.0). Non-specific binding sites were then blocked for 20 min using 5 % normal donkey serum in PBS.

For double immunofluorescence staining, spinal cord sections were incubated with primary rabbit antibodies against the five different DPRs (poly-GA, GP, GR, PR and PA) at a dilution of 1:100 together with TDP-43 (rat monoclonal TDP-43 Sigma Scientific (SIG-39850)) at a dilution of 1:100 overnight in a humid chamber at 4 °C. The DPR antibodies were obtained from Dr. Leonard Petrucelli and colleagues and were previously characterised [13]. After 3 washes with PBS, sections were incubated with anti-rabbit (Alexa Fluor 594) and anti-rat (Alexa Fluor 488) secondary antibodies for 1 h at room temperature. DAPI (Sigma) was used to counterstain nuclei. Sections were mounted in Fluorsave. Semi-quantitative and quantitative evaluation of DPR and TDP-43 pathology was performed using Zeiss Axiovert S100 microscope. Aggregates were also imaged using a Leica Confocal SP microscope.

### Semi quantitative and quantitative evaluation of pathology

The presence of DPR and TDP-43 inclusions were scored in the anterior horn of the spinal cord using a previously published semi-quantitative grading scale [14], in which the total number of immunoreactive inclusions, as well as the cytoplasmic inclusions (CI) and the intranuclear inclusions (NI), were rated as follows: 0 – Absent, 0.5 – one or two inclusions in the whole section, 1- very few, 2- occasional- easy to find and a few cells are affected, 3- moderate- many of the cells are affected, inclusions are easy to find, 4- numerous- nearly all of the cells are affected. Additionally, each section was re-analysed and the number of DPR and TDP-43 inclusions was counted manually in a systematic, blinded manner. The number of DPR aggregates was counted per section and averaged per case. Statistical analysis was performed with GraphPad Prism software (version 5.0).

## Results

### Appearance of DPR proteins in ALS spinal cords

**Fig. 1:**
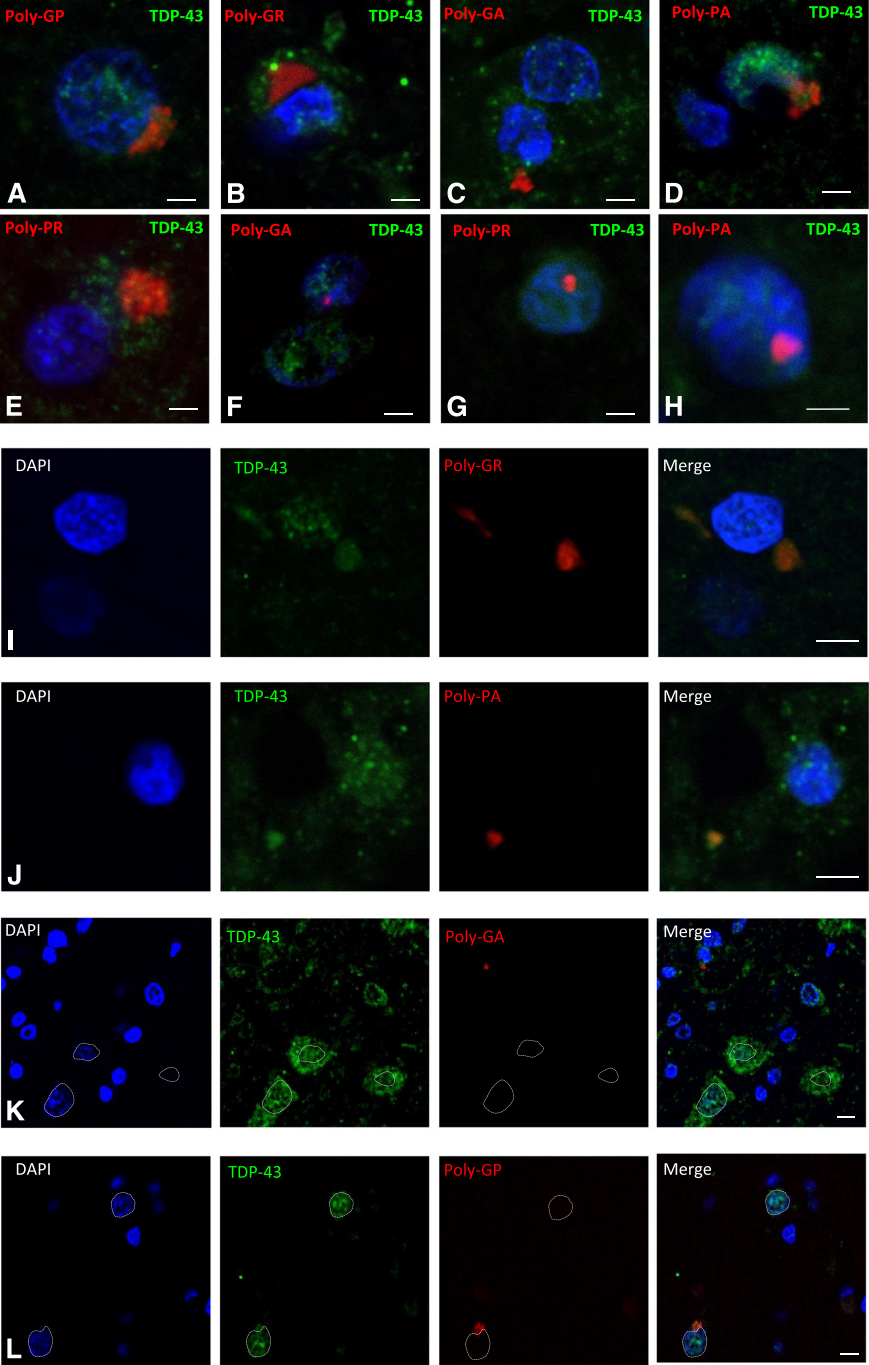
Representative images of DPR inclusions. **a**-**j** are representative immunofluorescence confocal images of DPR (red) and TDP-43 (green) (scale bar = 20 μm). **a**-**e** show cytoplasmic DPR inclusions, which are often perinuclear. **f**-**h** show nuclear DPR inclusions. **i** and **j** show evidence of DPR inclusions colocalizing with TDP-43. **k** and **l** are lower power images of the anterior horn showing the relative abundance of DPR and TDP-43 inclusions (scale bar = 50 μm). **l** shows a poly-GA inclusion, which is not in the motor neurons (dotted nucleus) that show extensive cytoplasmic TDP deposition. **k** shows a poly-GP inclusion within a motor neuron (identified by its large pale nucleus), which does not show cytoplasmic TDP-43 inclusions. Motor neurons were identified by their characteristic large pale nucleus (dotted line)

The appearance and subcellular location of each type of DPR inclusion within the spinal cord was recorded (Fig. 1a-h). Poly-GA aggregates were the most abundant DPR detected with cytoplasmic immunoreactive inclusions more common than intranuclear inclusions. This is consistent with previous studies in FTD and ALS [9]. Cytoplasmic inclusions were characteristically large, irregular and perinuclear whilst nuclear inclusions were much smaller and round. Poly-GP inclusions, the second most common DPR inclusion, were all cytoplasmic and in a perinuclear position (Fig. 1b). We observed very few cytoplasmic poly-GR inclusions and only one small intra-nuclear aggregate. Poly-PR cytoplasmic aggregates were also both nuclear and cytoplasmic (Fig. 1e, g). Similarly, we were able to identify a total of only seven poly-PA inclusions (six cytoplasmic and one nuclear; Fig. 1d, h) and four poly-PR inclusions (two cytoplasmic and two nuclear; Fig. 1e, g) inclusions in all the analyzed cases (Fig. 1d, h). No immunoreactive DPR aggregates were identified in any C9 negative ALS TDP-43 cases confirming the mutation-specificity of these inclusions and validating the antibodies. In a small number of cells that contained DPR aggregates, granular TDP-43 staining was seen in the cytoplasm or absent in the nucleus (Fig. 1) consistent with previous studies [9]. This pattern of TDP-43 staining was however much more common in neurons without DPR aggregates.

### All DPRs are rare and some very rare, but TDP-43 pathology was abundant and consistent

The frequency of each DPR inclusion was initially scored using a semi-quantitative system for each case (Table 2). DPRs translated from the sense strand (poly-GP, poly-GA and poly-GR) were more abundant than those translated solely from the antisense strand (poly-PR, poly-PA). Although poly-GA and poly-GP were the most abundant immunoreactive inclusions (scoring of 0.9 and 0.7, respectively) all DPRs were scored as either rare or very rare and there was not a single case that scored above 1.0 (rare) and no more than twelve inclusions of any DPR was observed in any of the cases analysed. No case scored above 0.5 (vary rare) for poly-GR, poly-PA and poly-PR inclusions and poly-GR and poly-PR inclusions were absent from several cases. The scoring of DPR aggregates within cases was highly consistent (poly-GA = 0.9 ± 0.1, poly-GP = 0.7 ± 0.1, poly-GR = 0.8 ± 0.21, poly-PA = 0.4 ± 0.20 and poly-PR = 0.3 ± 0.1). Of note, there was no significant difference in the scoring of TDP-43 inclusions for ALS cases lacking the *C9ORF72* mutation (2.8 ± 0.2) when compared to those carrying the *C9ORF72* mutation (2.9 ± 0.1). Thus, the presence of DPR inclusions in the spinal cord does not appear to be correlated with the abundance of TDP-43 inclusions.

**Table 2:** TDP-43 and DPR scoring

	TDP-43 agg.	Poly-GP			Poly-GA			Poly-GR			Poly-PR			Poly-PA			Diagnosis
case		Cyt.	Nuc.	Tot.	Cyt.	Nuc.	Tot.	Cyt.	Nuc.	Tot.	Cyt.	Nuc.	Tot.	Cyt.	Nuc.	Tot.	
1	3	0	0	0	0	0	0	0	0	0	0	0	0	0	0	0	ALS-TDP
2	3	0	0	0	0	0	0	0	0	0	0	0	0	0	0	0	ALS-TDP
3	2	0	0	0	0	0	0	0	0	0	0	0	0	0	0	0	ALS-TDP
4	3	0	0	0	0	0	0	0	0	0	0	0	0	0	0	0	ALS-TDP
5	3	0	0	0	0	0	0	0	0	0	0	0	0	0	0	0	ALS-TDP
MEAN ± SEM	2.8 ± 0.2																ALS-TDP
6	3	0.5	0	0.5	0	0	0	0.5	0	0.5	0	0	0	0.5	0	0.5	ALS-C9 + ve
7	3	0.5	0	0.5	1	0.5	1.5	0	0	0	0	0	0	0	0	0	ALS-C9 + ve
8	2	1	0	1	0.5	0	0.5	0.5	0	0.5	0	0	0	0.5	0	0.5	ALS-C9 + ve
9	3	1	0	1	1	0.5	1.5	0	0	0	0	0	0	0.5	0	0.5	ALS-C9 + ve
10	3	0.5	0	0.5	0.5	0.5	1	0.5	0	0.5	0	0	0	0	0	0	ALS-C9 + ve
11	3	1	0	1	1	0.5	1.5	0	0.5	0.5	0.5	0	0.5	0.5	0	0.5	ALS-C9 + ve
12	3	0.5	0	0.5	0.5	0	0.5	0.5	0	0.5	0.5	0.5	1	0	0	0	ALS-C9 + ve
13	3	1	0	1	1	0.5	1.5	0.5	0	0.5	0.5	0	0.5	0.5	0	0	ALS-C9 + ve
14	3	1	0	1	0.5	0	0.5	0	0	0	0	0	0	0	0	0	ALS-C9 + ve
15	3	0.5	0	0.5	1	0	1	0	0	0	0	0	0	0	0	0	ALS-C9 + ve
MEAN ± SEM	2.9 ± 1	0.7 ± 0.1	0.0	0.7 ± 0.1	0.7 ± 0.1	0.2 ± 0	0.9 ± 0.1	0.3 ± 0.1	0.0 ± 0.1	0.3 ± 0.1	0.1 ± 0.1	0.0 ± 0.1	0.2 ± 0.1	0.2 ± 0.1	0.0 ± 0.1	0.3 ± 0.1	

Scoring the frequency of TDP-43 and DPR inclusions in the spinal cord of *C9ORF72* mutation negative (1–5) and positive ALS cases (6–15). Cyt- cytoplasmic inclusion. Nuc- Nuclear inclusion. Scoring criteria: 0 – Absent 0.5 – one or two inclusions in whole section, 1- very few, 2- occasional- easy to find and a few cells are affected, 3- moderate- many of the cells are affected, inclusions are easy to find, 4- numerous- nearly all of the cells are affected

**Fig. 2:**
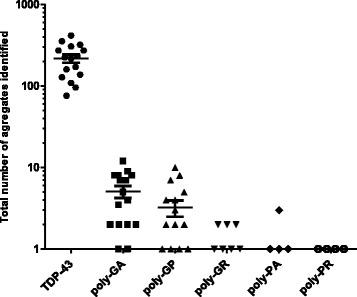
Frequency of TDP-43 and DPR inclusion per case, per section. The number of TDP-43 and DPR immunoreactive inclusions was counted for each case and normalised per spinal cord section. The graph shows the number of TDP-43 and the five different aggregates in order of abundance. There are many more TDP-43 aggregates than any DPR. Note: The Y axis scale is logarithmic scale. Horizontal bars show mean ± SEM

In order to provide greater detail, the number of TDP-43 aggregates in the anterior horn of the spinal cord in each case was quantified in representative sections to explore any correlation between the abundance of DPR and TDP-43 inclusions. An average of 122 ± 8 TDP-43 aggregates was identified per section per case, which is between 45- and 750- fold greater than the number of any DPRs inclusions (Additional file 1: Table S1). These results, graphically depicted in Fig. 2, demonstrate that the abundance of TDP-43 cytoplasmic inclusions vastly outnumbers the number of DPR inclusions, singly or collectively (note that the Y axis scale is a logarithmic scale).

### DPR and TDP-43 inclusions occasionally colocalize

Although all DPRs were rare or very rare, we did see TDP-43 colocalize with 4 of the 12 poly-GR inclusions and 2 of the 7 poly-PA inclusions (Fig. 1i, j). Interestingly, the poly-GR immunoreactive aggregates that did not colocalize were often surrounded by TDP-43 (Fig. 1b). None of the nuclear poly-GR and poly-PA intranuclear inclusions colocalized with TDP-43. We did not observe any poly-GP, poly-GA or poly-PR immunoreactive inclusions that colocalized with TDP-43 inclusions.

### DPRs are almost absent from motor neurons

ALS is characterised by the loss of lower motor neurons in the anterior horn of the spinal cord. Motor neurons are readily identified as they have large cell bodies and large pale nuclei by H&E staining and by DAPI for immunofluorescence. The total number of motor neurons was counted in order to assess the relative preservation of motor neurons between cases and identify any that contained TDP-43 and DPR immunoreactive inclusions. In total, of the 786 motor neurons analysed 268 (34 %) contained cytoplasmic TDP-43 inclusions (Fig. 1k), which is consistent with classical ALS pathology. In contrast, only one motor neuron contained a DPR inclusion, specifically a cytoplasmic poly-GP aggregate (0.1 %) (Fig. 1l). Occasionally we observed DPR and TDP-43 aggregates in the same cell but never in motor neurons.

## Discussion

The G_4_C_2_ repeat mutation in the *C9ORF72* gene is the most common cause of ALS and FTLD known to date. A leading mechanistic hypothesis is that neurotoxicity is due to the expression and aggregation of DPRs, particularly poly-GR and poly-PR that form intranuclear inclusions [15–17]. In this study, we sought to characterise the abundance, subcellular and cellular localization of immunoreactive inclusions for each DPR and TDP-43 in the anterior horn of the spinal cord in *C9ORF72* mutant positive and negative ALS cases.

Inclusions for all five DPRs were detected in the spinal cord of ALS-C9+ve cases but they were rare or very rare and it was uncommon to see DPR and TDP-43 inclusions in the same cell. Poly-GA inclusions, present in the cytoplasm and occasionally the nucleus, were the most common. Poly-GP inclusions were purely cytoplasmic, which is consistent with published reports [7–10,14,18,19]. Of the five DPRs, only poly-GR and poly-PA physically colocalized with TDP-43 inclusions, but this was a very rare event. The total number of TDP-43 inclusions in the spinal cord was between 45- and 750-fold higher than the number of DPR inclusions. We detected only one motor neuron that contained a poly-GP inclusion, whereas on average 34 % of motor neurons had cytoplasmic TDP-43 inclusions. Our results are consistent with those published using single staining techniques but are the first to use double labelling with TDP-43 and all five DPRs in the spinal cord of ALS cases [9]. We acknowledge that detection of DPRs by immunofluorescence imaging may be less sensitive than horse radish peroxidase (HRP) but these were abundant in the frontal cortex of our *C9ORF72* mutation positive cases (Additional file 2: Figure S1).

Several groups have studied DPR protein toxicity in various cellular models but these have generated conflicting results [15–17,20,21]. Two recent reports describe the greatest toxicity from poly-GR and poly-PR expression in cells [15,16] and the drosophila eye [22]. However, these models involve the overexpression of DPRs and no comparison was made with the burden of DPR deposition in human tissues. A recent study, in which 66 G_4_C_2_ repeats were expressed in the central nervous system of mice, has shown that nuclear RNA foci, DPR inclusions *and* TDP-43 pathology were present in the cortex and hippocampus of mice, suggesting that the repeat itself, possibly through foci formation, RAN translation or another mechanisms entirely, caused aberrant TDP-43 deposition [23]. We have demonstrated that DPR inclusions are very rare in ALS-C9+ve spinal cords, are infrequently associated with TDP-43 inclusions and are almost absent from motor neurons. On this basis we find no pathological evidence that DPR aggregation contributes to lower motor neuron degeneration in ALS-C9+ve cases.

## Conclusions

While we cannot entirely exclude the possibility that DPR aggregation and toxicity occurs at the sub-microscopic level, through toxic DPR oligomers, or that all motor neurons containing DPR inclusions were lost, this explanation seems unlikely as neurotoxic protein aggregates are detected in the affected neurons for all other neurodegenerative diseases. As the number of TDP-43 inclusions in ALS cases, with and without the *C9ORF72* mutation are nearly identical we can find no evidence that dipeptide repeat proteins are playing a pathogenic role in ALS-C9+ve cases.

## Additional files

Additional file 1: Table S1. Table showing the total numbers of TDP-43 and DPR aggregates per spinal cord section analysed. Additional file 2: Figure S1. Representative images of DPR inclusions in the frontal cortex of *C9ORF72* cases. Representative images of DPR aggregates in the frontal cortex of *C9ORF72* mutation positive cases used as a positive control. Dotted area indicates the zoomed in area. Arrows indicate the presence of DPR aggregates. (Scale bar = 50 μm).
