# Supplementary material for: Dipeptide repeat protein inclusions are rare in the spinal cord and almost absent from motor neurons in C9ORF72 mutant amyotrophic lateral sclerosis and are unlikely to cause their degeneration
Source: Acta Neuropathol Commun. 2015 Jun 25;3:38. doi: 10.1186/s40478-015-0218-y (PMC4479315; doi:10.1186/s40478-015-0218-y)
Supplement: Additional file 1: Table S1. — Table showing the total numbers of TDP-43 and DPR aggregates per spinal cord section analysed. [file 40478_2015_218_MOESM1_ESM.pdf]

Table S1: Quantification of TDP-43 and DPR aggregates in the anterior horn per section, per case

	TDP-43 agg.	Poly-GP			Poly-GA			Poly-GR			Poly-PR			Poly-PA			Diagnosis
case		Cyt.	Nuc.	Tot.	Cyt.	Nuc.	Tot.	Cyt.	Nuc.	Tot.	Cyt.	Nuc.	Tot.	Cyt.	Nuc.	Tot.	
1	NA	0	0	0	0	0	0	0	0	0	0	0	0	0	0	0	ALS-TDP
2	NA	0	0	0	0	0	0	0	0	0	0	0	0	0	0	0	ALS-TDP
3	NA	0	0	0	0	0	0	0	0	0	0	0	0	0	0	0	ALS-TDP
4	NA	0	0	0	0	0	0	0	0	0	0	0	0	0	0	0	ALS-TDP
5	NA	0	0	0	0	0	0	0	0	0	0	0	0	0	0	0	ALS-TDP
**MEAN±SEM**																	**ALS-TDP**
6	123	1	0	1	0	0	0	1	0	1	0	0	0	0.5	0	0.5	ALS-C9+ve
7	109	0.5	0	0.5	3	0.5	3.5	0	0	0	0	0	0	0	0	0.0	ALS-C9+ve
8	171	8	0	8	1	0	1	1	0	1	0	0	0	1.0	0	1.0	ALS-C9+ve
9	102	3	0	3.3	2.3	0.3	2.7	0	0	0	0	0	0	0.7	0.3	1.0	ALS-C9+ve
10	107	0.3	0	0.3	0.3	0.3	0.7	0.3	0	0.3	0	0	0	0	0	0.0	ALS-C9+ve
11-sacral	116	2.5	0	2.5	2	1.5	3.5	0	0	0	0	0	0	0.5	0	0.5	ALS-C9+ve
11- cervical	80	1.5	0	1.5	3	1.5	4.5	0	0.5	0.5	0.5	0	0.5	0	0	0.0	
12-Cervical	128	1	0	1	4	0	4	2	0	2	0	1	1	0	0	0.0	ALS-C9+ve
12-Lumbar	138	0	0	0	2	0	2	1	0	1	1	0	1	0	0	0.0	
13-Cervical	208	1	0	1	2.5	0	2.5	1	0	1	0	0	0	0.5	0	0.5	ALS-C9+ve
13-thoracic	136	3.5	0	3.5	2.5	1.5	4	1	0	1	0	0	0	0	0	0.0	
13-thoracic	112	2	0	2	4	2	6	0	0	0	0	0.5	0.5	0	0	0.0	
14-cervical	136	1	0	1	1	0	1	0	0	0	0	0	0	0	0	0.0	ALS-C9+ve
14-thorathic	76	2	0	2	1	0	1	0	0	0	0	0	0	0	0	0.0	
14-lumbar	96	4	0	4	2	0	2	0	0	0	0	0	0	0	0	0.0	
15	118	0.3	0	0.3	2.7	0	2.7	0	0	0	0	0	0	0	0	0.0	ALS-C9+ve
**MEAN±SEM**	**122±8**	**2±0.5**	**0.0**	**2±0.5**	**2.1±0.3**	**0.5±0.2**	**2.6±0.4**	**0.5±0.2**	**0.0±0.1**	**0.5±0.1**	**0.1±0.1**	**0.1±0.1**	**0.2±0.1**	**0.2±0.1**	**0.0±.01**	**0.2±0.1**	
