# Supplementary figures and images for: Dipeptide repeat protein inclusions are rare in the spinal cord and almost absent from motor neurons in C9ORF72 mutant amyotrophic lateral sclerosis and are unlikely to cause their degeneration
Source: Acta Neuropathol Commun. 2015 Jun 25;3:38. doi: 10.1186/s40478-015-0218-y (PMC4479315; doi:10.1186/s40478-015-0218-y)

Supplementary Figure 1

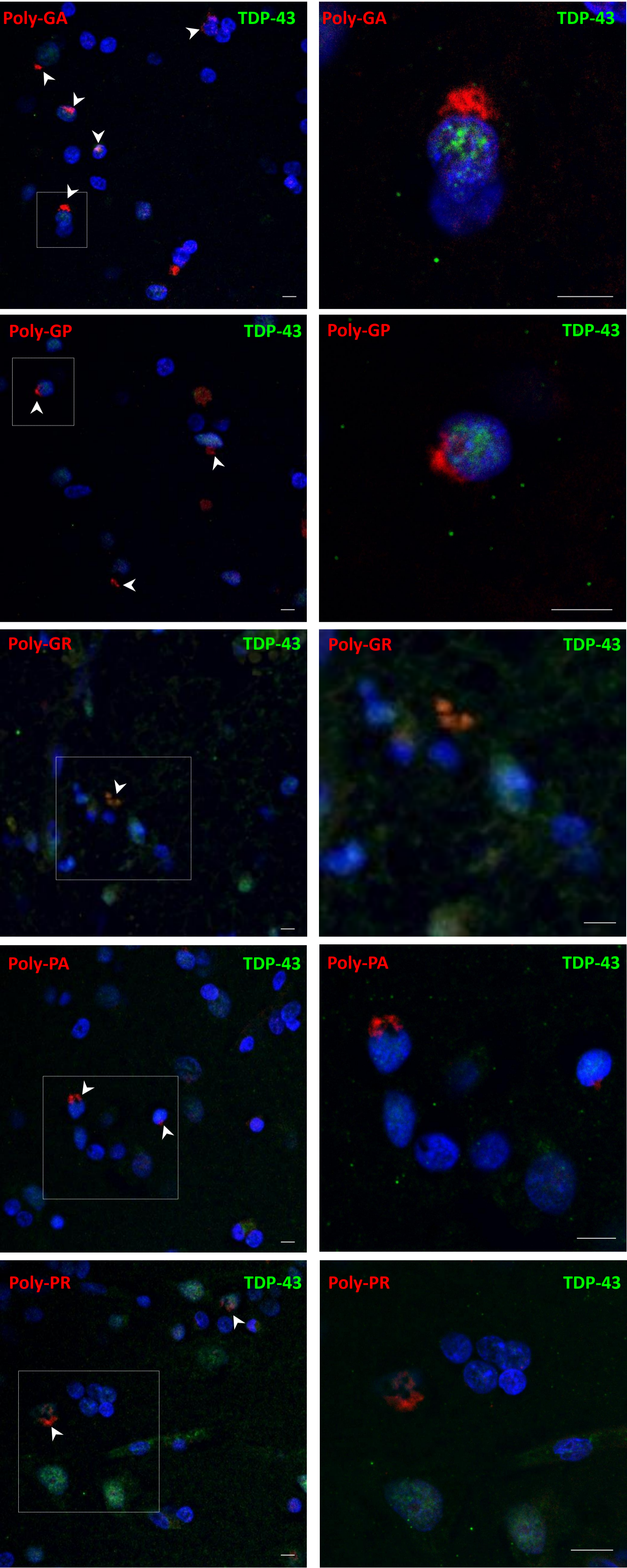

Supplement: Additional file 2: Figure S1. — Representative images of DPR inclusions in the frontal cortex of C9ORF72 cases. Representative images of DPR aggregates in the frontal cortex of C9ORF72 mutation positive cases used as a positive control. Dotted area indicates the zoomed in area. Arrows indicate the presence of DPR aggregates. (Scale bar = 50 μm). [file 40478_2015_218_MOESM2_ESM.pdf]
